# Comprehensive analysis of carotid triangle lymph node metastasis in papillary thyroid carcinoma

**DOI:** 10.3389/fendo.2026.1899033

**Published:** 2026-07-24

**Authors:** Yi Zhou, Zhixin Guo, Mingwei Liang, Jitian He, Ruixia Li, Yuan Hu, Xiangdong Xu, Wanna Chen

**Affiliations:** 1 Department of Thyroid Surgery, The First Affiliated Hospital of Sun Yat-sen University, Guangzhou, China; 2 Organ Transplant Center, The First Affiliated Hospital of Sun Yat-sen University, Guangzhou, China

**Keywords:** carotid triangle, lateral neck dissection, lymph node metastasis, papillary thyroid carcinoma, risk factor

## Abstract

**Background:**

Carotid triangle lymph nodes (CAT LN) represent a frequently overlooked region during lateral neck dissection (LND) in patients with papillary thyroid carcinoma (PTC), and their metastatic characteristics remain poorly defined. This study aimed to comprehensively evaluate the clinicopathological risk factors and metastatic patterns associated with CAT LN involvement.

**Methods:**

This single-center retrospective study analyzed 410 lateral neck dissections performed in 380 patients with PTC. The lateral neck side, paired with the ipsilateral thyroid lobe and ipsilateral central compartment, was used as the unit of analysis. Clinicopathological variables associated with CAT LN metastasis were evaluated using univariate and multivariate logistic regression analyses. The status of lymph node metastasis in other cervical regions was compared between CAT-positive and CAT-negative neck sides. Conditional probability analysis was further performed to explore predominant metastatic pathways according to tumor location.

**Results:**

CAT LN metastasis was observed in 174 of 410 dissected neck sides (42.4%). Multivariate analysis identified tumor size >2 cm (OR = 2.243, 95% CI: 1.431–3.547, P < 0.001), tumor location, and aspect ratio ≥1 (OR = 2.181, 95% CI: 1.280–3.802, P = 0.005) as independent predictors of CAT LN metastasis. Upper-pole tumors had a significantly higher risk (OR = 1.791, 95% CI: 1.145-2.816, P = 0.011), while lower-pole tumors had a lower risk (OR = 0.295, 95% CI: 0.147-0.559, P < 0.001) compared to middle-pole tumors. CAT-positive necks demonstrated significantly higher metastatic rates in levels II, III, and VI and had a greater total number of involved nodal levels (all P < 0.05), indicating a substantially higher metastatic burden across cervical lymph node regions. Conditional probability analysis revealed multiple potential metastatic putative pathways leading to the carotid triangle (CAT). Moreover, the lymphatic metastatic putative routes to the CAT were strongly correlated with primary tumor location: CAT LN metastasis in upper-pole tumors appeared to preferentially spread through the lateral cervical lymphatic pathway, whereas CAT involvement in middle-pole tumors was more likely to arise through upward dissemination from the central compartment.

**Conclusions:**

CAT LN metastasis is common among PTC patients undergoing LND and is associated with tumor size, tumor location, aspect ratio, and overall regional nodal burden. The metastatic pattern of CAT LN appears to vary according to tumor location, suggesting distinct lymphatic dissemination pathways. Increased awareness of this anatomical region may improve surgical completeness and optimize lateral neck dissection strategies in selected high-risk patients.

## Introduction

Papillary thyroid carcinoma (PTC) is the most common endocrine malignancy, and its incidence continues to rise globally (1). Cervical lymph node metastasis is a hallmark of PTC, occurring in a significant proportion of patients, and is a critical determinant of disease staging, recurrence risk, and the extent of surgery (2, 3). Therapeutic lateral neck dissection is generally performed for clinically evident lateral nodal disease, but the optimal assessment of subregional nodal areas remains an important surgical question (4).

**Figure 1:**
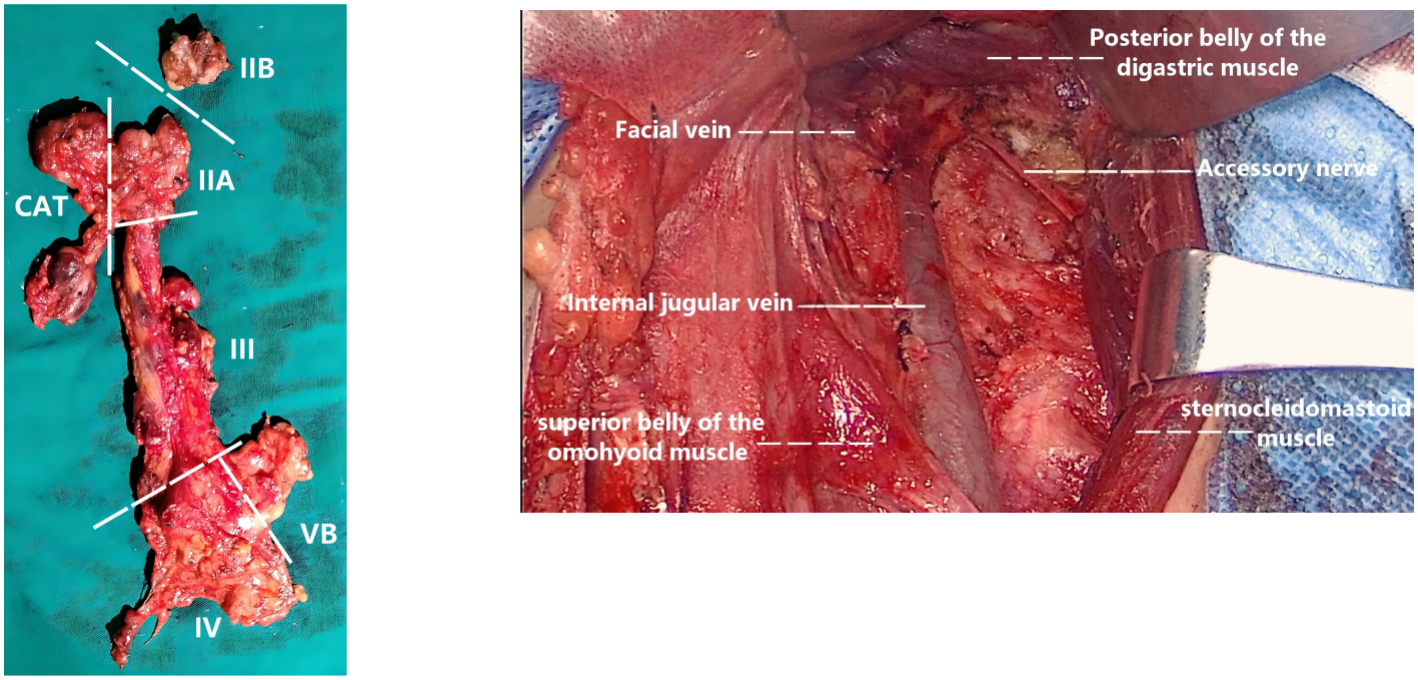
Surgical specimen and operative view showing the borders of the carotid triangle.

The carotid triangle (CAT) is an anatomically distinct region bounded anterolaterally by the anterior border of the sternocleidomastoid muscle, superiorly by the posterior belly of the digastric muscle, and medially by the superior belly of the omohyoid muscle (5). This region contains critical neurovascular structures, including the common carotid artery and its branches (internal and external carotid arteries), the internal jugular vein, the vagus nerve, the hypoglossal nerve, and the accessory nerve (6) (**Figure 1**).

Current neck-level classification systems define levels II-VI but do not designate the CAT as an independent nodal level (7, 8). Thus, CAT nodes may be removed en bloc during LND without separate pathological evaluation, limiting knowledge of their independent clinical significance. In the present study, the CAT was therefore treated as a distinct anatomical subregion and was dissected and submitted separately for pathological evaluation to investigate its independent clinical significance. While previous studies have extensively examined metastatic patterns and risk factors in the lateral cervical compartment, the specific metastatic behavior and clinical relevance of lymph nodes within the CAT remain poorly defined. This knowledge gap limits the optimization of surgical strategies and may contribute to incomplete lymph node clearance and residual disease in a subset of patients.

Therefore, this study aimed to: (1) identify clinicopathological risk factors for CAT LN metastasis; (2) characterize its association with other cervical node regions; and (3) explore tumor location-specific lymphatic dissemination patterns to the CAT.

## Methods

### Study design and patients

This was a single-center retrospective cohort study approved by the institutional ethics committee, with the requirement for informed consent waived because of its retrospective design. We retrospectively analyzed the medical records of patients with PTC who underwent thyroidectomy at The First Affiliated Hospital of Sun Yat-sen University between January 2019 and May 2025. The inclusion criteria were: (1) age ≥18 years; (2) newly diagnosed PTC without prior treatment; (3) total thyroidectomy combined with central neck dissection (CND) and LND; and (4) lymph nodes from the CAT submitted separately for pathological examination. The exclusion criteria were: (1) no malignant nodule in the thyroid lobe ipsilateral to the analyzed LND side; (2) previous neck surgery, radiation exposure, or thermal ablation; (3) family history of thyroid cancer; (4) incomplete medical records; and (5) concomitant malignant tumors.

### Unit of analysis and side-specific assignment

All included patients underwent total thyroidectomy. The analytical unit was not a thyroid lobectomy but a lateral neck side. For each analyzed LND side, tumor characteristics were assigned from the ipsilateral thyroid lobe. If more than one malignant nodule was present in that lobe, the largest malignant nodule was defined as the index lesion for ultrasonographic and pathological variables. For patients who underwent bilateral LND, the left and right neck sides were initially included as separate side-level observations, each paired with the corresponding ipsilateral thyroid lobe.

For the side-specific attribution of level VI status, the central compartment was divided into midline central nodes, including prelaryngeal and pretracheal nodes, and lateralized central nodes, including left and right paratracheal nodes. For each analyzed neck side, level VI metastasis was considered positive if metastasis was identified in either the midline central nodes or the ipsilateral paratracheal central compartment. Specifically, for left lateral neck dissections, level VI status was defined according to the presence of metastasis in the midline central nodes and/or the left central compartment; for right lateral neck dissections, level VI status was defined according to the presence of metastasis in the midline central nodes and/or the right central compartment. If both the midline central nodes and the corresponding ipsilateral central compartment were negative, level VI was classified as negative for that neck side. In bilateral cases, this rule was applied separately to the left and right neck sides.

### Surgical treatment and pathological evaluation

Preoperative assessment included physical examination, high-resolution ultrasonography (The aspect ratio was defined as the anteroposterior diameter divided by the transverse diameter of the index malignant thyroid nodule on preoperative ultrasonography.), and contrast-enhanced computed tomography (CT). All patients underwent total thyroidectomy with CND and LND. During LND, lymph nodes from levels IIA, IIB, III, IV, VB, and the CAT were dissected and submitted as separate specimens for pathological examination. Because a sizeable proportion of level IV and level V specimens were submitted as a combined specimen, levels IV and V were analyzed together as IV+V in the regional and conditional probability analyses. All pathological specimens were reviewed by experienced pathologists and staged according to the UICC/AJCC TNM system.

### Statistical analysis

Statistical analyses were performed according to the types and distributions of variables. Continuous variables were presented as mean ± standard deviation (SD) for normally distributed data or median with interquartile range (IQR) for non-normally distributed data. Normality of continuous variables was assessed using the Shapiro–Wilk test. Categorical variables were summarized as counts and percentages. Comparisons between groups were performed using the independent-samples t-test for normally distributed continuous variables, the Mann–Whitney U test for non-normally distributed variables, and the Chi-square test or Fisher’s exact test for categorical variables, as appropriate. The primary outcome was CAT LN metastasis at the neck-side level.

Univariable binary logistic regression was used for the univariate screen of candidate variables. Tumor location was modeled as a multi-category variable with middle-pole tumors as the reference category. Candidate variables included sex, age category (<45 vs. ≥45 years), ethnicity, diabetes, hypertension, hyperthyroidism, Hashimoto thyroiditis, TSH, FT3, FT4, TgAb, TPOAb, Tg, tumor size (>2 vs. ≤2 cm), capsular invasion, tumor location, tumor composition, aspect ratio, blood-flow pattern, echogenicity, lymph-node calcification pattern, and margin. Variables with P < 0.05 in univariate analysis were included in multivariate binary logistic regression models to identify independent risk factors associated with carotid triangle lymph nodes (CAT LN) metastasis. Both unadjusted and adjusted odds ratios (ORs) with 95% confidence intervals (CIs) were reported.

Because bilateral neck sides from the same patient may share tumor biology, operative conditions, and pathological processing, a sensitivity analysis restricted to unilateral LND cases was performed. Specifically, all patients who underwent bilateral LND were excluded, and the same variable-selection procedure used in the primary analysis was repeated: candidate variables were first evaluated using univariable binary logistic regression, and variables with P < 0.05 were then entered into the multivariable binary logistic regression model in the unilateral-only cohort. (n = 350 neck sides).

Regional nodal co-involvement was compared between CAT-positive and CAT-negative neck sides. The metastatic burden was defined as the number of involved nodal regions among level II, level III, level IV+V, and level VI, ranging from 0 to 4. Differences in metastatic burden between CAT-positive and CAT-negative neck sides were assessed using the Mann-Whitney U test and accompanied by rank-biserial correlation as an effect-size measure.

Conditional probabilities were calculated as follows: P(CAT+ | region+) was defined as the probability of CAT LN metastasis among neck sides with metastasis in a specified nodal region, whereas P(CAT+ | region-) was defined as the probability of CAT LN metastasis among neck sides without metastasis in that region. The absolute risk difference (Delta Risk) was calculated as P(CAT+ | region+) - P(CAT+ | region-). Delta Risk was used descriptively to quantify the difference in CAT LN metastasis probability according to the presence or absence of metastasis in each nodal region. All statistical tests were two-tailed, and a P-value < 0.05 was considered statistically significant. Statistical analyses were performed using Python and GraphPad Prism.

## Results

### Baseline characteristics

A total of 380 patients with PTC were included, accounting for 410 dissected neck sides, including 30 bilateral and 350 unilateral dissections. The mean age was 38.84 ± 11.18 years (range, 18–77 years), and female patients predominated (62.1%). The cohort was predominantly Han Chinese (368/380, 96.8%). Detailed baseline characteristics are presented in **Table 1**.

**Table 1:** Baseline characteristics of PTC patients.

Variable	Original PTC patient (N = 380)
Gender (male)	144 (37.8%)
Age (years)	38.84 ± 11.18
Ethnicity (Han)	368 (96.8%)
Diabetes (yes)	14 (3.6%)
Hypertension (yes)	25 (6.5%)
Hyperthyroidism (yes)	18 (4.7%)
Hashimoto’s thyroiditis (yes)	82 (21.5%)
TSH (uIU/mL)	1.73 (1.13, 2.48)
FT3 (pmol/L)	5.00 (4.58, 5.50)
FT4 (pmol/L)	11.69 (10.2, 12.8)
TgAb (IU/mL)	0.25 (0.017, 6.28)
TPOAb (IU/mL)	0.9 (0.35, 18.2)
Tg (ng/mL)	18.70 (5.54, 56.48)
Bilateral LND	30 (7.8%)

TSH, thyroid-stimulating hormone; FT3, free triiodothyronine; FT4, free thyroxine; TgAb, thyroglobulin antibodies; TPOAb, thyroid peroxidase antibodies; Tg, thyroglobulin; LND, lateral neck dissection.

### Risk factors for CAT LN metastasis

Histopathological examination revealed CAT LN metastasis in 174 of 410 neck sides (42.4%). To identify risk factors associated with CAT LN metastasis, univariate and multivariate logistic regression analyses were performed (**Table 2**). Univariate analysis demonstrated that tumor size>2 cm, tumor location in the upper pole, and aspect ratio ≥1 were significant risk factors. In multivariate analysis, all three factors remained independent predictors. Tumor size >2 cm was associated with a more than two-fold increased risk (OR = 2.243, 95% CI: 1.431–3.547, P < 0.001), as was an aspect ratio ≥1 (OR = 2.181, 95% CI: 1.280–3.802, P = 0.005). Using the middle pole as a reference, upper-pole tumors conferred a significantly higher risk (OR = 1.791, 95% CI: 1.145-2.816, P = 0.011), while lower-pole tumors had a markedly lower risk (OR = 0.295, 95% CI: 0.147-0.559, P < 0.001).

**Table 2:** Univariable and multivariable logistic regression analyses of factors associated with carotid triangle lymph node metastasis.

Variable	CAT− (n = 236)	CAT+ (n = 174)	Univariable analysis		Multivariable analysis	
			OR (95% CI)	P value	Adjusted OR (95% CI)	P value
Gender						
Male	90	72	Reference		—	—
Female	146	102	0.873 (0.585–1.304)	0.507	—	—
Age						
<45 years	166	117	Reference		—	—
≥45 years	70	57	1.155 (0.756–1.762)	0.503	—	—
Ethnicity						
Han	228	169	Reference		—	—
Non-Han	8	5	0.843 (0.251–2.573)	0.768	—	—
Diabetes						
No	226	168	Reference		—	—
Yes	10	6	0.807 (0.270–2.217)	0.684	—	—
Hypertension						
No	221	161	Reference		—	—
Yes	15	13	1.190 (0.543–2.573)	0.659	—	—
Hyperthyroidism						
No	226	166	Reference		—	—
Yes	10	8	1.089 (0.408–2.820)	0.860	—	—
Hashimoto's thyroiditis						
No	185	139	Reference		—	—
Yes	51	35	0.913 (0.560–1.476)	0.713	—	—
TSH, uIU/mL	1.74 (1.06–2.49)	1.77 (1.27–2.57)	1.059 (0.992–1.131)	0.085	—	—
FT3, pmol/L	5.01 (4.60–5.50)	4.93 (4.57–5.50)	1.076 (0.814–1.422)	0.607	—	—
FT4, pmol/L	11.97 (10.43–13.05)	11.26 (9.98–12.59)	1.066 (0.985–1.153)	0.115	—	—
TGAB, IU/mL	0.48 (0.05–12.63)	0.52 (0–3.34)	1.000 (0.999–1.001)	0.479	—	—
TPOAB, IU/mL	0.90 (0.39–17.38)	0.96 (0.35–21.62)	1.000 (0.999–1.001)	0.935	—	—
TG, ng/mL	19.33 (3.15–60.79)	18.61 (6.94–55.12)	1.000 (0.999–1.001)	0.941	—	—
Tumor size						
≤2 cm	175	104	Reference		Reference	
>2 cm	51	70	1.931 (1.270–2.947)	0.002	2.243 (1.431–3.547)	<0.001
Capsular invasion						
No	157	112	Reference		—	—
Yes	79	62	1.100 (0.729–1.660)	0.650	—	—
Tumor location						
Middle	103	75	Reference		Reference	
Upper	69	85	1.692 (1.097–2.621)	0.018	1.791 (1.145–2.816)	0.011
Lower	64	14	0.300 (0.152–0.562)	<0.001	0.295 (0.147–0.559)	<0.001
Composition						
Solid	220	162	Reference		—	—
Non-solid	16	12	1.019 (0.460–2.202)	0.963	—	—
Aspect ratio						
<1	57	27	Reference		Reference	
≥1	179	147	1.734 (1.053–2.913)	0.034	2.181 (1.280–3.802)	0.005
Blood flow						
Poor	130	90	Reference		—	—
Normal	9	10	1.605 (0.623–4.192)	0.324	—	—
Rich	97	74	1.102 (0.735–1.652)	0.638	—	—
Echogenicity						
Hypoechoic	197	153	Reference		—	—
Non-hypoechoic	39	21	0.693 (0.392–1.227)	0.209	—	—
Lymph node calcification						
No calcification	28	19	Reference		—	—
Microcalcification	147	111	1.078 (0.575–2.021)	0.814	—	—
Macrocalcification	26	18	1.020 (0.442–2.356)	0.963	—	—
Other calcification	35	26	1.392 (0.576–3.364)	0.463	—	—
Margin						
Clear	96	62	Reference		—	—
Unclear	140	112	1.239 (0.828–1.862)	0.300	—	—

Values are presented as n for categorical variables or median (IQR) for continuous variables.

Univariable analyses were performed using binary logistic regression for each candidate variable. Tumor location was entered as a categorical variable with middle-pole tumors as the reference category.

Multivariable analysis included tumor size, tumor location, and aspect ratio. ORs in the multivariable columns are adjusted estimates.

OR, odds ratio; CI, confidence interval; CAT, carotid triangle; TSH, thyroid-stimulating hormone; FT3, free triiodothyronine; FT4, free thyroxine; TGAB, thyroglobulin antibody; TPOAB, thyroid peroxidase antibody; TG, thyroglobulin.

After excluding the 30 bilateral dissection patients, a unilateral-only sensitivity analysis was performed in 350 unilateral neck sides. The same variable-selection procedure used in the primary analysis was repeated. The independent factors identified in the unilateral-only cohort were consistent with those in the primary analysis: tumor size >2 cm, upper-pole tumor location, and aspect ratio ≥1 were associated with increased odds of CAT LN metastasis, whereas lower-pole tumor location was associated with decreased odds (**Supplementary Table 1**).

### Regional nodal co-involvement

**Figure 2:**
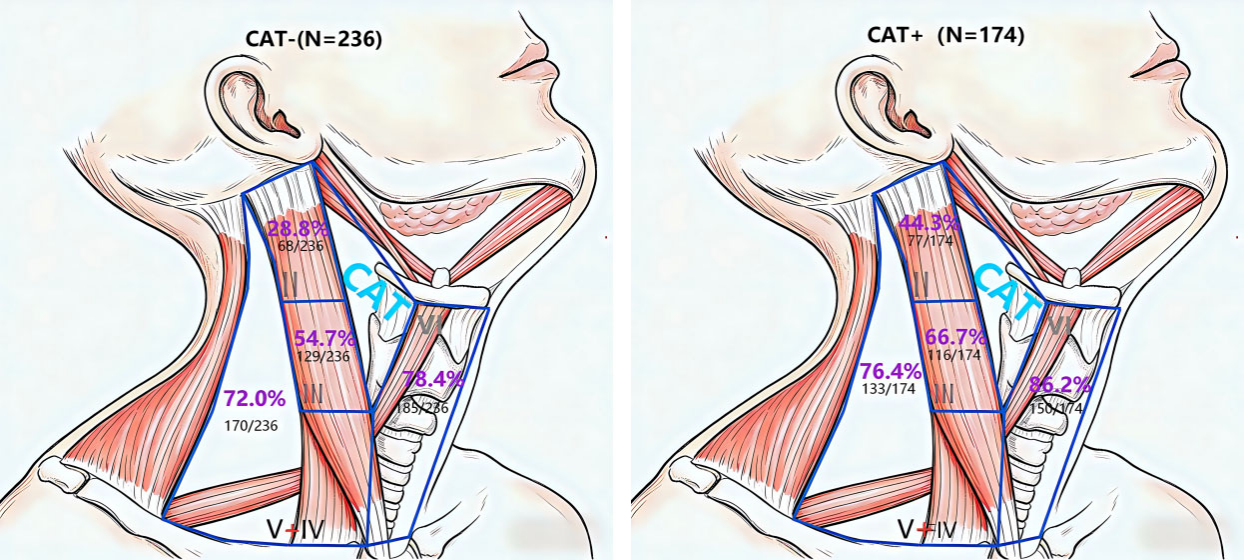
Comparison of metastatic rates across cervical lymph node levels between CAT LN-negative and CAT LN-positive neck sides.

The metastatic rates in all cervical compartments were higher in the CAT-positive group than in the CAT-negative group (**Figure 2**). Specifically, level II metastasis was observed in 44.3% (77/174) of neck sides in the CAT-positive group, compared with 28.8% (68/236) in the CAT-negative group. Similarly, level III metastasis occurred in 66.7% (116/174) of CAT-positive neck sides, which was higher than the 54.7% (129/236) observed in CAT-negative patients. Central compartment (level VI) metastasis was also more frequent in the CAT-positive group (86.2%, 150/174) than in the CAT-negative group (78.40%, 189/236). These differences were statistically significant for levels II, III, and VI (all P < 0.05). In addition, metastasis to the combined level IV+V specimen was more common in the CAT-positive group (76.4%, 133/174) than in the CAT-negative group (72.0%, 170/236), although the magnitude of this difference was relatively smaller (P > 0.05).

### Metastatic burden and CAT involvement

**Figure 3:**
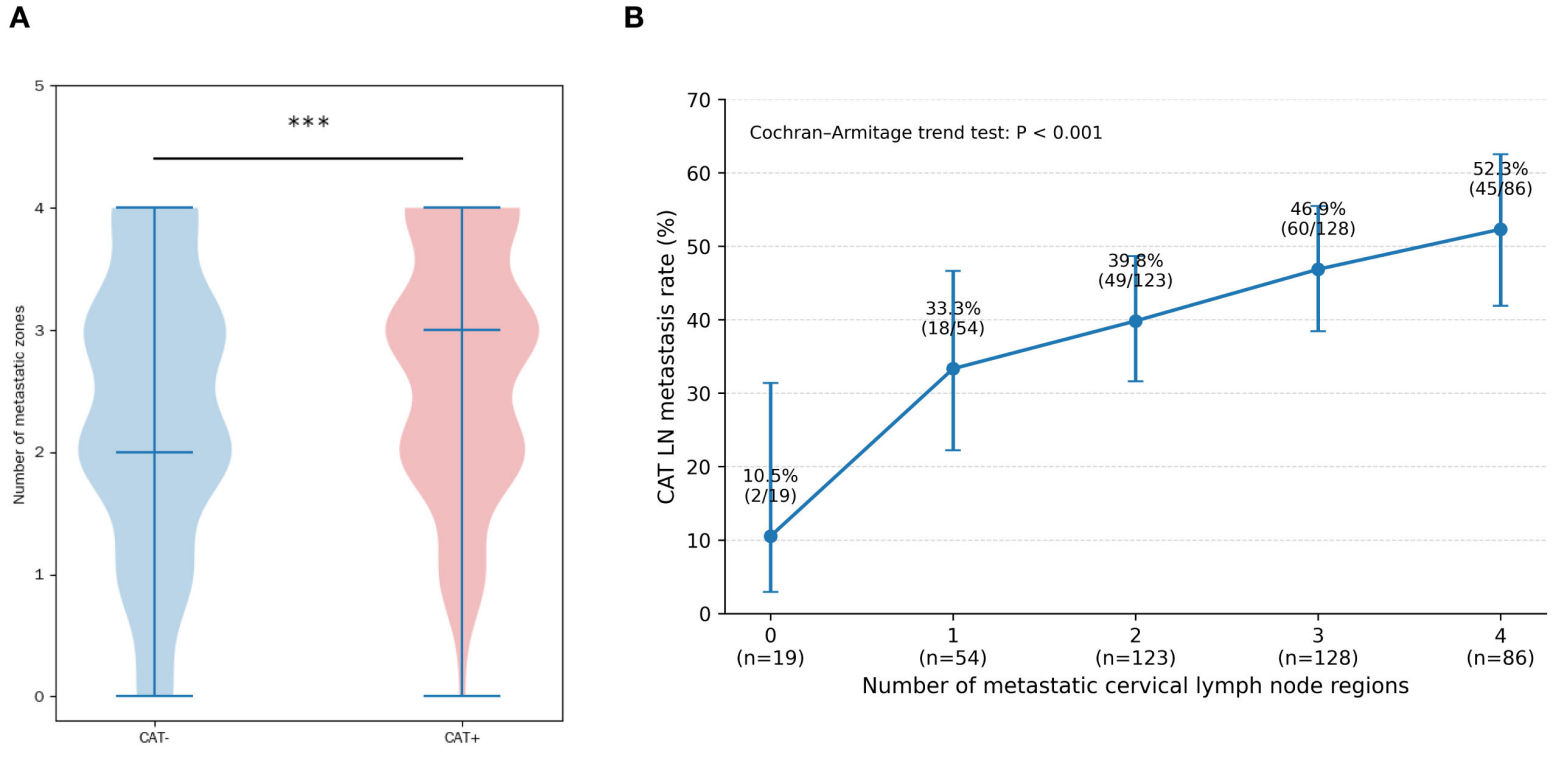
**(A)** Association between the number of metastatic lymph node regions and CAT LN metastasis. **(B)** Probability of CAT LN metastasis according to the number of metastatic cervical lymph node regions. ***P < 0.001

To further evaluate the relationship between carotid triangle involvement and metastatic burden, the number of metastatic cervical lymph node regions was compared between CAT-positive and CAT-negative groups (**Figure 3A**). The number of involved cervical lymph node regions was greater in CAT-positive neck sides than in CAT-negative neck sides (median, 3 vs. 2; Mann–Whitney U = 24414.5, P < 0.001), with a small effect size (rank-biserial correlation = 0.189). Further analysis demonstrated a progressive increase in the probability of CAT LN metastasis with increasing numbers of metastatic regions. The rate of CAT LN metastasis increased from 10.5% in neck sides with no metastatic regions to 33.3%, 39.8%, 46.9%, and 52.3% in patients with one, two, three, and four metastatic regions, respectively (**Figure 3B**). Cochran–Armitage trend testing confirmed an increasing trend (P < 0.001). Notably, the largest absolute increase was observed between zero and one metastatic region, whereas subsequent increases were more gradual, suggesting that the presence of any cervical nodal metastasis may represent an important threshold associated with CAT involvement rather than a strictly linear dose-response pattern.

### Conditional probability analysis

Conditional probability analysis demonstrated that the presence of metastasis in specific cervical lymph node regions markedly increased the probability of carotid triangle (CAT) involvement, suggesting that these regions may function as preferential intermediate stations in the metastatic cascade toward the CAT (**Table 3**). Overall, the baseline probability of CAT metastasis was 42.4%. Among all nodal regions, level II metastasis produced the largest probability difference, increasing CAT involvement from 36.6% to 53.1% (ΔRisk = 16.5%), followed by level VI (ΔRisk = 12.8%) and level III (ΔRisk = 12.1%).

**Table 3:** Conditional probability and risk difference of CAT metastasis according to cervical lymph node involvement and tumor location.

Tumor location	P(CAT+) (%)	Region	P(CAT+ | region+) (%)	P(CAT+ | region-) (%)	Delta Risk (%)
Overall	42.4	VI	44.8 (150/335)	32.0 (24/75)	12.8
		II	53.1 (77/145)	36.6 (97/265)	16.5
		III	47.3 (116/245)	35.2 (58/165)	12.1
		IV+V	43.8 (133/303)	38.3 (41/107)	5.5
Upper pole	55.1	VI	59.5 (69/116)	42.1 (16/38)	17.4
		II	66.2 (45/68)	46.5 (40/86)	19.7
		III	58.4 (59/101)	49.1 (26/53)	9.3
		IV+V	61.1 (66/108)	41.3 (19/46)	19.8
Middle pole	42.1	VI	44.8 (69/154)	25.0 (6/24)	19.8
		II	50.9 (29/57)	38.0 (46/121)	12.9
		III	46.7 (50/107)	35.2 (25/71)	11.5
		IV+V	41.3 (55/133)	44.4 (20/45)	-3.1
Lower pole	17.9	VI	18.5 (12/65)	15.4 (2/13)	3.1
		II	15.0 (3/20)	19.0 (11/58)	-4.0
		III	18.9 (7/37)	17.1 (7/41)	1.8
		IV+V	19.3 (12/62)	12.5 (2/16)	6.8

CAT, carotid triangle. P(CAT+) indicates the overall probability of CAT LN metastasis. P(CAT+ | region+) indicates the probability of CAT LN metastasis when metastasis is present in the specified region. P(CAT+ | region-) indicates the probability when metastasis is absent in that region. Delta Risk = P(CAT+ | region+) - P(CAT+ | region-).

When stratified by tumor location, distinct location-specific patterns were observed. In upper-pole tumors, the largest ΔRisk values were observed in levels II (19.7%) and IV+V (19.8%), indicating a predominance of lateral cervical dissemination pathways. In contrast, among middle pole tumors, level VI metastasis produced the largest difference (ΔRisk = 19.8%), suggesting a putative central compartment–associated pathway to CAT involvement. Lower pole tumors demonstrated a relatively low baseline probability of CAT metastasis (17.9%), with generally small probability differences across regions.

## Discussion

This study provides a dedicated investigation of carotid triangle lymph node (CAT LN) metastasis in papillary thyroid carcinoma (PTC), based on a relatively large surgical cohort in which the CAT region was separately submitted for pathological evaluation. By focusing on the CAT as an anatomically distinct yet clinically underrecognized subregion, the present study offers new insight into its metastatic characteristics and potential surgical relevance.

Several important findings emerged from the present study. First, we identified tumor size >2 cm, aspect ratio ≥1, and tumor location as independent predictors of CAT LN metastasis. Second, compared with CAT-negative neck sides, CAT-positive neck sides showed significantly higher metastatic rates across all nodal regions and a greater number of involved nodal levels, indicating a strong association between CAT involvement and overall lymph node metastatic burden. Third, conditional probability analysis suggested that lymphatic drainage pathways to the carotid triangle (CAT) may differ according to primary tumor location. However, these findings should be interpreted as associative and hypothesis-generating rather than as evidence of definitive lymphatic drainage pathways.

In the present cohort, CAT LN metastasis was identified in 42.4% of dissected neck sides. The CAT is not designated as an independent level in conventional neck dissection classification systems (7, 8). Therefore, separate pathological reporting of this region is uncommon, and CAT-specific evidence remains limited. In clinical practice, insufficient surgical experience, limited specialized training, and the absence of systematic evaluation of this anatomical region may contribute to under-recognition of CAT LN. Evidence from other non-standard cervical nodal subregions, particularly lymph nodes between the sternocleidomastoid and sternohyoid muscles, suggests that nodal tissues located outside conventional level-based definitions may be overlooked if they are not deliberately identified and submitted separately (9, 10). Although these regions are anatomically distinct from the CAT, they illustrate the broader clinical importance of careful subregional assessment during neck dissection. Our findings thus reinforce the importance of meticulous dissection of these less scrutinized areas, particularly the carotid triangle.

Anatomically, the carotid triangle is part of the lateral cervical compartment. Regarding risk factors, our results are generally consistent with the established literature on lateral neck metastasis in PTC (11–14). The strong influence of tumor location on CAT metastasis risk is particularly noteworthy. The finding that upper-pole tumors carry the highest risk aligns with the known propensity of these lesions to metastasize to the lateral neck, likely via lymphatic channels that accompany the superior thyroid vessels (15). Conversely, the markedly reduced risk associated with lower-pole tumors (OR = 0.295) indicates that lower-pole tumors had a lower observed probability of CAT co-involvement. These observations further underscore the importance of tumor location as a determinant of metastatic behavior and suggest that more detailed investigation of tumor location–specific lymphatic drainage patterns is warranted in patients with PTC.

Our study further confirmed a distinct cumulative effect: the risk of CAT LN metastasis rose gradually as the burden of metastatic lymph node regions increased. Specifically, the incidence of CAT LN metastasis increased steadily from 10.5% in patients without involvement of other lymph node regions to 52.3% in those with metastatic lesions in four lymph node regions. The most prominent increase appeared between zero and one involved nodal region, whereas subsequent increases across additional regions were smaller. From a surgical perspective, these findings support careful inspection and clearance of the CAT region in patients with clinically or pathologically evident cervical nodal metastasis, particularly when other risk factors such as upper-pole location, larger tumor size, or suspicious ultrasonographic features are present.

The thyroid gland possesses a large and highly interconnected lymphatic drainage system. Cai et al. proposed that lymph nodes are interconnected by lymphatic vessels, forming an integrated lymphatic drainage network spanning the entire cervical region (16). Therefore, lymph node metastasis does not follow a single linear pathway; rather, lymph nodes within the same region may have multiple potential routes of metastasis. Our conditional probability results are compatible with this network concept: CAT metastasis did not disappear when any single nodal region was negative, suggesting that multiple nonexclusive patterns of nodal co-involvement may exist.

The conditional probability analysis further suggested that the association between CAT LN involvement and metastasis in other cervical nodal regions may vary according to primary tumor location. In upper-pole tumors, level II and level IV+V involvement showed the largest probability differences, suggesting a predominance of lateral compartment dissemination. In middle-pole tumors, level VI involvement showed the largest probability difference, potentially reflecting upward extension from central compartment involvement toward the CAT region. In contrast, lower-pole tumors had a lower baseline probability of CAT LN metastasis and showed only small probability differences across nodal regions. This finding is consistent with previous studies, which have demonstrated that lymphatic drainage from the upper pole follows the superior thyroid artery and vein toward the upper jugulodigastric lymph node basin, whereas middle-pole tumors preferentially metastasize to level VI (central compartment) nodes (17–19). The conditional probability analysis was cross-sectional and associative, and no lymphatic mapping technique was performed in the present study. Future studies incorporating lymphatic mapping techniques, such as sentinel lymph node biopsy or indocyanine green fluorescence imaging, are required to validate whether these putative patterns reflect true lymphatic dissemination routes. From a clinical perspective, these findings suggest that surgeons should maintain awareness of possible CAT involvement in patients with upper-pole tumors and suspicious lateral cervical nodes, as well as in patients with middle-pole tumors and central compartment metastasis.

## Limitations

This study has several limitations. First, the study was retrospective and carried out at a single center, so some degree of selection bias may be present. The cohort was also predominantly Han Chinese (96.8%), limiting generalizability to more ethnically diverse populations. Second, 30 patients underwent bilateral LND and contributed two neck-side observations. Although a unilateral-only sensitivity analysis was added to assess robustness, the two sides in bilateral cases may share tumor biology, operative decision-making, and pathological processing; therefore, complete statistical independence cannot be assumed in the full side-level analysis. Third, the study included only patients who underwent LND with separate CAT pathological evaluation, and the decision to perform bilateral dissection may reflect surgeon judgment or perceived risk rather than true bilateral biological disease. Fourth, because a sizeable proportion of patients had their level IV and V lymph nodes examined pathologically as a combined specimen, we had to analyze these two levels together. This is a structural limitation of the conditional probability analysis, because levels IV and V may have different anatomical relationships and location-specific lymphatic associations. Pooling may particularly obscure potential lower-pole patterns. Fifth, we did not evaluate long-term oncological outcomes such as recurrence and survival; therefore, the independent prognostic significance of CAT LN metastasis remains undetermined. Sixth, prospective multicenter studies with long-term follow-up are required to further validate these findings and clarify the prognostic significance of CAT LN metastasis.

## Conclusions

Carotid triangle lymph node metastasis is a prevalent and predictable event in PTC, and it is closely linked to primary tumor characteristics and the overall burden of regional disease. Our findings introduce a topography-based paradigm for lymphatic dissemination. In high-risk patients—those with upper-pole tumors, larger cancers, or aggressive ultrasound features—thorough dissection of the carotid triangle merits particular attention. We need prospective multi-institutional studies to validate these predictors and to clarify whether CAT involvement independently affects recurrence and survival.

## Data Availability

The raw data supporting the conclusions of this article will be made available by the authors, without undue reservation.

## Supplementary material

Supplementary Table 1 Univariable and multivariable logistic regression analyses of factors associated with CAT LN metastasis in the unilateral-only cohort. Unilateral-only sensitivity cohort: 350 neck sides after excluding all bilateral LND patients (CAT-positive = 143; CAT-negative = 207).
