# Supplementary material for: Comprehensive analysis of carotid triangle lymph node metastasis in papillary thyroid carcinoma
Source: Front Endocrinol (Lausanne). 2026 Jul 24;17:1899033. doi: 10.3389/fendo.2026.1899033 (PMC13447421; doi:10.3389/fendo.2026.1899033)
Supplement: Supplementary file 1 [file Table1.docx]

**Supplementary Table S1. Univariable and multivariable logistic regression analyses of factors associated with CAT LN metastasis in the unilateral-only cohort**

Unilateral-only sensitivity cohort: 350 neck sides after excluding all bilateral LND patients (CAT-positive = 143; CAT-negative = 207).

| **Variable** | **Comparison** | **Unadjusted OR (95% CI)** | **P value** | **Adjusted OR (95% CI)** | **P value** |
| --- | --- | --- | --- | --- | --- |
| Female sex | Female vs male | 1.081 (0.694–1.684) | 0.731 | — | — |
| Age group | 1 vs 0 | 1.026 (0.648–1.625) | 0.913 | — | — |
| Ethnicity | 1 vs 0 | 1.214 (0.363–4.056) | 0.753 | — | — |
| Diabetes | Yes vs no | 0.471 (0.125–1.773) | 0.266 | — | — |
| Hypertension | Yes vs no | 1.002 (0.417–2.412) | 0.996 | — | — |
| Hyperthyroidism | Yes vs no | 1.167 (0.449–3.034) | 0.751 | — | — |
| Hashimoto’s thyroiditis | Yes vs no | 1.009 (0.605–1.683) | 0.973 | — | — |
| TSH | Per 1 uIU/ml increase | 1.041 (0.970–1.117) | 0.263 | — | — |
| FT3 | Per 1 pmol/l increase | 1.096 (0.816–1.473) | 0.542 | — | — |
| FT4 | Per 1 pmol/l increase | 1.117 (1.018–1.225) | 0.019 | — | — |
| TGAB | Per 1 IU/ml increase | 1.001 (0.999–1.002) | 0.258 | — | — |
| TPOAB | Per 1 IU/ml increase | 0.999 (0.998–1.001) | 0.462 | — | — |
| TG | Per 1 ng/ml increase | 1.000 (0.999–1.001) | 0.776 | — | — |
| Tumor size >2 cm | >2 cm vs ≤2 cm | 1.796 (1.143–2.822) | 0.011 | 2.121 (1.302–3.456) | 0.003 |
| Capsular invasion | Yes vs no | 1.223 (0.785–1.905) | 0.373 | — | — |
| Tumor location | Upper vs middle | 1.755 (1.095–2.814) | 0.019 | 1.828 (1.122–2.976) | 0.015 |
| Tumor location | Lower vs middle | 0.300 (0.145–0.623) | 0.001 | 0.291 (0.138–0.612) | 0.001 |
| Composition | 1 vs 0 | 1.002 (0.417–2.412) | 0.996 | — | — |
| Aspect ratio ≥1 | ≥1 vs <1 | 1.796 (1.042–3.095) | 0.035 | 2.134 (1.193–3.816) | 0.011 |
| LN calcification | 1 vs 0 | 1.237 (0.609–2.513) | 0.556 | — | — |
| LN calcification | 2 vs 0 | 1.071 (0.373–3.074) | 0.898 | — | — |
| LN calcification | 3 vs 0 | 1.320 (0.533–3.266) | 0.548 | — | — |
| LN calcification | 4 vs 0 | 1.576 (0.607–4.091) | 0.350 | — | — |
| Margin unclear | Yes vs no | 1.171 (0.757–1.813) | 0.478 | — | — |
| Blood flow | 1 vs 0 | 0.781 (0.253–2.413) | 0.668 | — | — |
| Blood flow | 2 vs 0 | 0.824 (0.264–2.574) | 0.738 | — | — |
| Non-low echogenicity | Yes vs no | 0.704 (0.386–1.282) | 0.251 | — | — |
